# Measurable Residual Disease in Adult Acute B-Lymphoblastic Leukemia: Methods, Guidelines, and Emerging Actionability at Ultra-Low-Level

**DOI:** 10.3390/cancers18091331

**Published:** 2026-04-22

**Authors:** Abeer Yaseen, Enas Abusalim, Mohamad Harb, Zaid Sarhan, Yazan Talab, Nazmi Kamal, Fareed Barakat, Nidal Al-Masri, Ayman Saad, Zaid Abdel Rahman

**Affiliations:** 1 Department of Internal Medicine, King Hussein Cancer Center, Amman 11941, Jordan; 2 School of Medicine, Al-Balqa’a Applied University, Salt 19117, Jordan; 3 Department of Cellular Therapy and Applied Genomics, King Hussein Cancer Center, Amman 11941, Jordan; 4 Department of Pathology and Laboratory Medicine, King Hussein Cancer Center, Amman 11941, Jordan; 5 Oncology Center, King Faisal Specialist Hospital and Research Center, Riyadh 11211, Saudi Arabia; 6 School of Medicine, University of Jordan, Amman 11941, Jordan

**Keywords:** measurable residual disease, acute lymphoblastic leukemia, next-generation sequencing, minimal residual disease monitoring, allogeneic hematopoietic cell transplantation, immunotherapy, risk stratification, MRD-guided therapy

## Abstract

Measurable residual disease (MRD) is the most powerful predictor of relapse and long-term survival in adult acute lymphoblastic leukemia (ALL), consistently outperforming traditional clinical and cytogenetic risk factors. The advent of high-sensitivity next-generation sequencing (NGS) capable of detecting MRD at 10^−6^ has transformed monitoring, reclassifying a substantial proportion of patients previously deemed negative by multiparameter flow cytometry (MFC) or quantitative PCR (qPCR), and revealing clinically relevant disease persistence at ultra-low levels. This review synthesizes current MRD detection platforms and their clinical applications across frontline therapy, allogeneic hematopoietic cell transplantation, and relapsed/refractory disease with specific focus on B-ALL. We integrate the 2024 European LeukemiaNet (ELN) and the 2025 US expert panel recommendations, highlighting important differences in preferred methodologies and decision thresholds. Particular attention is given to the emerging role of early deep NGS negativity in guiding transplant deferral among selected standard-risk patients, including some with Ph+ ALL treated with chemotherapy-free regimens, and to the challenges of interpreting persistent low-level positivity (10^−4^–10^−6^). Despite technological advances, key questions remain: when should deeper detection trigger therapeutic escalation, and how should discordance between modalities, peripheral blood monitoring, and subtype-specific variability be interpreted? Addressing these issues through prospective validation, platform harmonization, and broader global access will be essential to ensure that increasing sensitivity translates into evidence-based, equitable clinical benefit.

## Simple Summary

Acute lymphoblastic leukemia (ALL) is a blood cancer that affects adults, and even after treatment, tiny amounts of cancer cells can remain, increasing the risk of relapse. This review explains how doctors measure these leftover cells (called measurable residual disease or MRD) using advanced tests like flow cytometry, PCR, and next-generation sequencing. We discuss new guidelines from experts in Europe and the US, which help decide when to intensify treatment, like using immunotherapy or transplants. The goal is to help patients live longer without over-treating them. By understanding ultra-low levels of MRD, doctors can make better decisions, but more research is needed to ensure these tests are accessible worldwide and truly improve outcomes.

## 1. Introduction

Over the past decade, the therapeutic landscape of adult acute lymphoblastic leukemia (ALL) has changed dramatically. Pediatric-inspired multi-agent intensive chemotherapy backbones, combined with the integration of targeted immunotherapies, including blinatumomab (CD19 bispecific T-cell engager), inotuzumab ozogamicin (anti-CD22 antibody-drug conjugate), and CD19-directed chimeric antigen receptor (CAR) T-cell therapies, as well as potent tyrosine kinase inhibitors (TKIs) in Philadelphia chromosome–positive (Ph+) have collectively increased complete remission (CR) rates to over 90% in many contemporary protocols [1,2,3,4,5]. Despite these advantages, long term survival in adults remain inferior to that observed in children, and relapse continues to represent the principal cause of treatment failure. The persistence of subclinical disease below the threshold of morphologic detection has emerged as the dominant biological explanation for this discrepancy [6,7].

Measurable residual disease (MRD), previously minimal residual disease, refers to the quantifiable presence of leukemic cells at levels as low as 10^−4^ to 10^−6^ that are undetectable by conventional microscopy [8]. In adults, MRD has evolved from a biologic marker into a central determinant of risk stratification and therapeutic decision-making in adult ALL, consistently outperforming conventional factors such as age, white blood cell count at diagnosis, cytogenetics, and molecular abnormalities in multivariable analyses across large cohorts and meta-analyses [9,10,11].

The recognition of MRD’s prognostic impact has driven a paradigm shift toward risk-adapted, MRD-guided therapy. Both the 2024 European LeukemiaNet (ELN) recommendations [5] and the 2025 United States (US) expert panel consensus [12] recommend standardized MRD assessment at defined timepoints, and MRD persistence frequently prompts therapeutic escalation, including immunotherapy or allogeneic hematopoietic cell transplantation (allo-HCT). These guidelines reflect mature data from pivotal trials, and underscore MRD as a surrogate endpoint increasingly accepted in clinical trials [11].

However, the increasing sensitivity of MRD testing has introduced new complexity. While deeper MRD detection improves relapse prediction, the clinical actionability of ultra-low-level positivity remains incompletely defined. Discordance between modalities, variability in peripheral blood (PB) versus bone marrow (BM) sensitivity [13], the impact of immunotherapy-induced antigen modulation [14], and the emergence of lineage plasticity [15] challenge uniform interpretation. Moreover, ELN and US expert panels differ in their preferred methodologies and thresholds, reflecting ongoing uncertainty regarding optimal integration of these tools into routine practice. Questions also persist regarding cost, global accessibility, and the risk of overtreatment when increasingly sensitive assays detect biologic persistence without clear evidence that intervention improves survival [16,17].

This review provides an updated, clinician-oriented synthesis of MRD assessment in adult ALL with a focus on B-ALL. We summarize current detection modalities and their technical characteristics, review contemporary clinical data across frontline, transplant, and relapsed/refractory settings. Particular attention is given to areas of evolving evidence, including ultra-deep MRD detection, modality discordance, peripheral blood monitoring, and management of low-level persistence. Through this framework, we aim to clarify how advances in MRD technology can be integrated into evidence-based clinical decision-making.

## 2. Methodology

This narrative review was structured to align with the quality criteria of the Scale for the Assessment of Narrative Review Articles (SANRA) [18], including clear justification of importance and aims, transparent description of the literature search, appropriate referencing, critical appraisal of evidence, and presentation of key data and endpoints.

A structured search of PubMed (MEDLINE), Embase, and the Cochrane Library was performed for English-language publications from January 2000 through January 2026 to capture the evolution of MRD assessment in adult acute lymphoblastic leukemia—from early flow cytometry and PCR studies to high-sensitivity next-generation sequencing (NGS) and contemporary guideline-driven strategies. Search terms included combinations of “measurable residual disease,” “minimal residual disease,” “acute lymphoblastic leukemia,” “adult ALL,” “flow cytometry,” “PCR,” “ddPCR,” “next-generation sequencing,” “clonoSEQ,” and “MRD-guided therapy.” Reference lists of key trials, meta-analyses, and international consensus guidelines (including ELN, US expert panel, EuroFlow, and EuroMRD) were manually reviewed to identify additional relevant studies

Eligible studies included phase I–III clinical trials, cohort studies, meta-analyses, and consensus guidelines evaluating MRD detection methods, reporting thresholds, timing of assessment, and clinical outcomes in adult ALL. Abstracts were included when considered high impact and were cross-referenced with full publications where available. Editorials and reports lacking direct relevance to MRD methodology or clinical application were excluded.

Data were synthesized qualitatively and organized into thematic sections addressing detection platforms, MRD in frontline therapy, transplant-related decision-making, relapsed or refractory disease, and areas of evolving evidence.

## 3. MRD Detection Modalities: What They Measure, What They Miss, and When They Matter

Accurate MRD assessment in adult ALL depends on the detection of leukemia-specific features that distinguish residual blasts from regenerating hematopoiesis. In clinical practice, three principal modalities are employed: Multiparameter Flowcytometry (MFC), quantitative polymerase chain reaction (qPCR), and next-Generation Sequencing (NGS) (Table 1). Rather than competing technologies, these methods interrogate different biological dimensions of residual disease; phenotypic, transcript-based, and clonotypic, leading to differences in analytic sensitivity, interpretive vulnerabilities, and clinical utility.

Rather than viewing them hierarchically, it is more useful to understand what each modality measures, where it performs optimally, and under what circumstances it may mislead.

### 3.1. Multiparameter Flow Cytometry (MFC)

MFC detects residual leukemic blasts through aberrant immunophenotypic signatures, defined either by leukemia-associated immunophenotypes (LAIPs) or standardized “different-from-normal” (DfN) strategies using fixed multicolor panels (e.g., EuroFlow 8–12-color systems) [19,20,21]. This approach enables rapid, accessible MRD evaluation without requiring patient-specific assay development, making it particularly valuable in resource-limited or urgent settings.

In routine practice, analytic sensitivity approximates 10^−4^ (0.01%), while optimized next-generation flow (NGF) protocols reaching 10^−5^ in experienced centers. NGF refers to standardized, high-sensitivity MFC approaches that incorporate optimized antibody panels, standardized sample processing, and quality control measures to improve reproducibility and sensitivity [22]. Conventional manual interpretation remains limited by its time-consuming nature and susceptibility to interpreter-dependent variability. Artificial intelligence (AI), with its capacity to perform repetitive and complex analytical tasks with high consistency, offers a promising solution to these challenges [23,24,25]. In a meta-analysis (4373 patients), with a pooled AUC of 0.954 (*p* < 0.001), sensitivity of 0.998, substantial reductions in analysis time (often >50%), and a clear association between AI-defined MRD negativity and improved overall survival (OS) and relapse-free survival [RFS) [26].

Sensitivity depends on specimen quality (preferably a first-pull bone marrow aspirate processed promptly), cellularity, and operator expertise [27]. The 10^−4^ threshold remains the historical benchmark embedded in most guideline-based treatment algorithms. PB monitoring remains substantially less sensitive and is not recommended for standard MRD surveillance.

Importantly, MFC-MRD negativity is lineage-specific rather than leukemia-specific as it relies on surface and cytoplasmic antigen expression rather than genotype rendering it vulnerable to therapeutic modulation. CD19-directed therapies [blinatumomab and CD-19 directed CAR-T) frequently cause antigen downregulation or complete loss [14,28]; with CD19-negative relapse occurring in roughly 20–30% of cases after blinatumomab and 20–40% after CAR-T therapy [28,29,30]. Similarly, emerging evidence suggests that CD22 modulation under selective therapeutic pressure may further complicate MRD assessment. CD22-targeted therapy with inotuzumab ozogamicin can drive the emergence or expansion of CD22^dim^/negative leukemic subclones, sometimes present at low levels prior to treatment, leading to antigen escape and potential underestimation of residual disease when CD22 is used as a monitoring marker. This phenotypic shift reflects antigen escape rather than true disease clearance and may lead to underestimation of residual disease when CD22 is used as a monitoring marker [31]. Collectively, these findings reinforce the need for dynamic, therapy-adapted MFC panels and caution against reliance on single antigen targets for MRD evaluation.

A more complex challenge arises following targeted immunotherapy is lineage plasticity. International analyses, including Project EVOLVE, document myeloid lineage switch “LS” after CD19-directed therapy (75 patients), especially in KMT2A-rearranged and BCR::ABL1-positive ALL, typically within 1–1.5 months after CD19-directed therapy, and is associated with a dismal median survival of only 4–4.8 months. These cases illustrate bidirectional lymphoid-to-monocytoid transdifferentiation despite preserved IG rearrangements [15,32,33].

To address these challenges, the EuroFlow consortium developed expanded 12-color BCP-ALL panels incorporating “CD19-negative” markers (e.g., CD22, CD24 and CD34) and exclusion markers (CD3, CD7), improving lineage definition in post-CD19 settings and enabling simultaneous assessment of CD22 expression to guide inotuzumab candidacy [20]. Standardization and harmonization of MFC-MRD have also been led by other major groups, including the Children’s Oncology Group (COG), UKALL [34], GMALL [35], and Russian protocols. These efforts employ redundant B-lineage markers (such as CD22, CD24, and intracellular CD79a) to maintain sensitivity after CD19-directed immunotherapy [36]. Currently, specific guidelines for assessment of CD22 expression by flow cytometry are not available [31]. In cases of suspected relapse after immunotherapy, especially in KMT2A-rearranged disease, it is advisable to test for myeloid markers even after loss of B-cell antigens. High-risk patients with KMT2A rearrangements require particularly close monitoring, even when in morphologic remission, using orthogonal methods combining MFC and molecular techniques.

The clinical relevance of MFC-based MRD assessment is well established. A large systematic review and meta-analysis by Bassan et al. (2019) [9] specifically in adult precursor B-cell ALL (23 studies, 5979 patients) confirmed the strong prognostic impact of MRD negativity, mostly within 3 months post-induction). Patients achieving MRD negativity had significantly better relapse-free survival (HR 2.34, 95% CI 1.91–2.86) and overall survival (HR 2.19, 95% CI 1.63–2.94) compared with MRD-positive patients. These benefits were consistent across Ph+ and Ph− subgroups, disease stages, detection methods, and sensitivity thresholds.

In summary, MFC remains the most widely accessible MRD platform and the historical benchmark for clinical decision-making. However, its dependence on surface/cytoplasmic antigen expression renders it vulnerable to immunotherapy-induced modulation and lineage plasticity. In the current therapeutic landscape, MFC is indispensable not only for MRD quantification but also for phenotypic characterization that molecular methods cannot provide.

### 3.2. Real-Time Quantitative Polymerase Chain Reaction (qPCR)

qPCR-based MRD monitoring targets defined molecular lesions and is widely used in adult B-ALL. Two distinct strategies are employed: detection recurrent fusion transcripts (e.g., *BCR::ABL1*, *E2A-PBX1*, *KMT2A*, *CRLF2*) (present in 40% of B-ALL patients), and patient-specific immunoglobulin/T-cell receptor (IG/TR) gene rearrangements (present in >80–90% of B-ALL patients) via allele-specific oligonucleotide (ASO) strategies.). These approaches differ in applicability, technical requirements, and clinical utility, with routine sensitivities of 10^−4^ to 10^−5^ being routinely achievable [37].

Fusion transcript monitoring is particularly central in Ph+ ALL, where *BCR::ABL1* quantification provides standardized molecular tracking (p210 on international scale; p190 as *BCR::ABL1/ABL1* ratio) [38]. Early molecular response is highly informative: achievement of complete molecular remission [CMR) at approximately three months strongly predicts favorable long-term survival and may allow some patients to avoid allo-HCT [39]. It is also essential for *BCR::ABL1* or *KMT2A* cases, particularly when immunophenotypic shifts or lineage switch compromise MFC [40,41].

At higher transcript levels (>0.01%), *BCR::ABL1* PCR-MRD shows high correlation with high-sensitivity MFC-MRD (r = 0.7801, concordance 88%; MFC detects MRD in 82.9% of such samples). However concordance declines at ultra-low levels, where persistent PCR positivity often does not represent true ALL MRD [42].

In Ph+ ALL, however, low-level persistent *BCR::ABL1* transcripts frequently reflect clonal hematopoiesis or a CML-like biology rather than viable leukemic disease due to the expansion of benign or pre-malignant clones carrying BCR::ABL1 that do not drive ALL relapse. In GRAAPH-2014 (de novo Ph+ ALL, 264 patients, treated with nilotinib + chemotherapy), 38% of patients exhibited BCR::ABL1 persistence linked to clonal hematopoiesis, with no survival benefit from allo-HCT in these cases (3-year OS 70% vs. 72% for MRD-negative; HR 1.05, *p* = 0.82) [43]. Recent 2024–2025 studies confirm no adverse survival impact in NGS-negative but PCR-positive cases [44,45]. These findings highlight that molecular detectability does not uniformly equate to leukemic viability and highlights the importance of orthogonal testing (preferably with NGS) before escalating therapy or proceeding to allo-HCT in deep responders.

Recent technological advances aim to improve the precision of PCR-based MRD detection. Digital droplet PCR (ddPCR) offers absolute quantification without a standard curve and superior low-level sensitivity [46]. In conventional qPCR, very low MRD signals that are detectable but fall below the reliable quantitative range of the assay are classified as “positive-not-quantifiable” (POS-NQ or PNQ). These ambiguous results create a clinically problematic gray zone, as they cannot be confidently assigned a precise MRD value for risk stratification or therapeutic decisions. In adult Philadelphia-negative B-ALL, one prospective analysis reported a reduction from 14% with conventional qPCR to 2.6% with ddPCR (*p* = 0.003) [47], while also enabling quantification in additional low-level samples. Similar improvements have been observed in other cohorts, including Ph+ ALL (46% of cases quantifiable (*p* < 0.0001)) [48] and mixed pediatric/adult series (where ddPCR resolved 83% of qPCR PNQ cases) [49], while also allowing quantification in additional low-level samples and improving concordance with NGS (87–92% between ddPCR and NGS vs. 52–57% for qPCR) [50].This improvement enhances MRD risk stratification, particularly at very low disease levels. Reflecting these advances, the 2024 EuroMRD update integrates ddPCR into quality assurance (QA), introducing refined low-level categories (“MRD low positive, <QR”) re-classifiable by ddPCR or NGS to minimize uncertainty [51].

When recurrent fusions are absent, ASO-qPCR targeting IG/TR rearrangements provides an alternative, biologically specific MRD marker [38,52]. In paired analyses of Ph+ B-ALL, concordance between *BCR::ABL1* PCR and IG/TR ASO-qPCR in bone marrow approximates 70%, with discordance most pronounced at low MRD levels [53]. Its implementation remains labor-intensive (4–6 weeks for assay development) and susceptible to clonal evolution, with potential for false negatives [54].

Sampling considerations further influence assay performance. In B-ALL, MRD levels are typically 1–3 logs lower in peripheral blood than in bone marrow, supporting marrow-based assessment for qPCR and MFC [55].

In summary, qPCR provides highly specific molecular MRD detection and remains an essential tool in molecularly defined ALL subtypes, particularly Ph+ disease, where it reports MRD as a log reduction from baseline or normalized ratio. Nevertheless, at very low transcript levels, interpretation is complex due to issues such as clonal hematopoiesis, indeterminate (POS-NQ) results, and clonal evolution. For these reasons, PCR-based MRD monitoring is often complemented by orthogonal methods such as flow cytometry or next-generation sequencing in contemporary clinical practice.

### 3.3. High-Throughput Next-Generation Sequencing (HT-NGS)

NGS interrogates IG/TR rearrangements via multiplex amplification and deep sequencing, tracking multiple clonotypes from diagnosis. With adequate DNA input, analytic sensitivity reaches 10^−6^ or lower; a 1–2 log improvement over prior methods [56,57].

The ClonoSEQ assay (Adaptive Biotechnologies) is the only FDA-cleared NGS-based MRD platform for B-ALL in BM, routinely achieving 10^−6^ sensitivity [58]. Unlike ASO-qPCR, it avoids clone-bias, reducing false negatives from marker loss due to clonal evolution, and enables continuous quantification and multi-clone tracking, improving detection of early molecular relapse [59].

Clinical studies in adult ALL demonstrate that NGS frequently detects residual disease not identified by MFC. NGS detects disease in up to 46% of MFC-negative cases, with early 10^−6^ negativity linked to >90–95% relapse-free survival in select cohorts [60,61]. However, the clinical actionability of ultra-low levels (10^−4^ to 10^−6^) remains unproven in prospective trials.

Discordance between NGS and transcript-based monitoring is particularly relevant in Ph+ B-ALL. In a retrospective analysis of 81 patients, 32% showed discordance, with 31% of NGS MRD-negative cases remaining PCR-positive for *BCR::ABL1*; importantly, no relapses occurred among these PCR+/NGS− patients (median follow-up > 5 years). NGS negativity more accurately predicted long-term RFS than PCR status alone (5-year RFS 84% vs. 73% for PCR+/NGS− vs. PCR−/NGS−, *p* = 0.84) [62]. Similarly, in a larger retrospective cohort (187 patients overall; 80 patients with paired early assessments), 23% of NGS-negative patients had detectable BCR::ABL1 by PCR, with no significant impact on outcomes: 3-year cumulative incidence of relapse (CIR) was 20% for NGS−/PCR− vs. 19% for NGS−/PCR+ (*p* = 0.81), RFS 74% vs. 78% (*p* = 0.59), and OS 88% vs. 91% (*p* = 0.68). Outcomes remained similar when stratified by PCR positivity level (0.01–0.1% vs. ≥0.1%; 3-year OS 93% vs. 88%, *p* = 0.65) [63]. These discrepancies are frequently attributable to non-leukemic clonal hematopoiesis rather than viable residual disease. Consequently, caution is warranted when NGS is negative, but PCR remains positive at low levels. In such cases the 2025 US guidelines support delaying or omitting allo-HCT in deep responding patients, prioritizing NGS-based IG/TR MRD over fusion transcript monitoring for risk stratification and transplant decisions.

NGS also offers potential advantages for less invasive monitoring strategies. Studies demonstrate reasonable BM–PB concordance in ALL, particularly when BM is negative or low-level, suggesting PB as a less invasive monitoring option once deep BM negativity is confirmed. However, PB levels are consistently 1–3 logs lower than BM, and discordance is frequent (PB negative while BM positive), limiting PB as a standalone tool for routine surveillance [13,64].

The 2025 US expert panel explicitly recommends BM MRD monitoring until negativity is documented using a high-quality sample, after which PB monitoring with NGS may be considered; either alone or alternating with periodic BM assessments, to reduce the frequency of invasive procedures, particularly in patients with low pretest probability of positivity. In contrast, ELN guidelines maintain BM as the standard sample for surveillance.

Reflecting accumulating evidence, the 2025 US expert panel recommends NGS as the preferred method for MRD-guided decision-making in B-ALL because of its superior sensitivity and relapse discrimination compared with MFC or qPCR. The 2024 ELN guidelines mention NGS primarily for molecular subtypes (e.g., Ph-like fusions/mutations) but do not yet endorse it as routine for IG/TR MRD, favoring qPCR/MFC for most cases.

Implementation barriers include cost, 7–10-day turnaround, diagnostic material quality, bioinformatics, background noise false positives, limited T-ALL validation, and DNA input needs. However, NGS’s ability to track clonal evolution and detect MRD below the traditional 10^−4^ threshold positions it as a transformative tool in precision management of adult ALL.

### 3.4. Comparative Strengths, Limitations, and Clinical Integration of MRD Modalities

**Figure 1:**
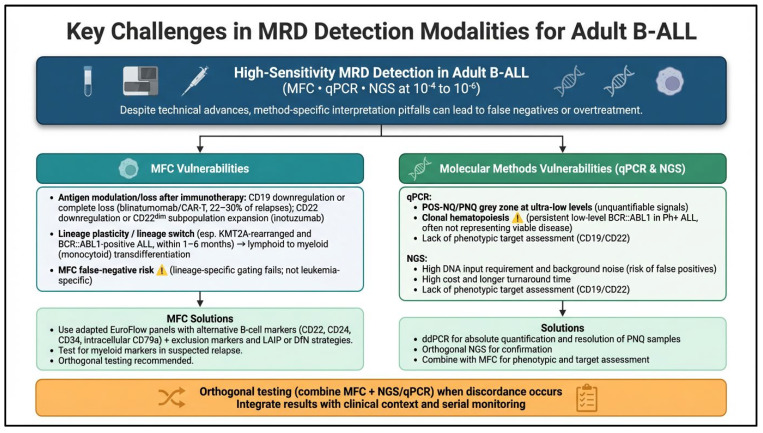
Key challenges and solutions in MRD detection modalities for adult B-ALL.

Although the three modalities can achieve overlapping analytic sensitivities, their complementary strengths make orthogonal use particularly valuable in complex cases (Figure 1). MFC provides rapid phenotypic information and unique insight into therapeutic target expression in the immunotherapy era, while molecular methods (qPCR and NGS) offer greater specificity and sensitivity for tracking clonal evolution and ultra-low disease burden. Two phenomena frequently complicate low-level MRD interpretation. POS-NQ/PNQ represents a technical gray zone of conventional qPCR that ddPCR or NGS can often resolve, whereas clonal hematopoiesis, most commonly seen with low-level *BCR::ABL1* in Ph+ ALL, reflects benign clones rather than viable leukemic disease and often carries no adverse prognostic impact when NGS is negative. The 2024 ELN and 2025 US expert panel recommendations further reflect these differences in thresholds and sampling strategies. The ELN adopts a more conservative approach, using a 10^−4^ cutoff as the primary trigger for ‘molecular failure’ and escalation (including allo-HCT), largely relying on MFC or qPCR. In contrast, the US panel recommends intervention for any detectable MRD (particularly by high-sensitivity NGS down to 10^−6^) provided there is high confidence it represents true disease, and supports PB NGS monitoring once deep BM negativity is confirmed to reduce invasive procedures. In practice, the optimal strategy frequently involves combining modalities, particularly after immunotherapy or in high-risk disease, to balance sensitivity, specificity, and actionable phenotypic data.

## 4. Clinical Implications of MRD in the Treatment of Adult B-ALL

MRD assessment in adult B-ALL serves two main purposes. Time-point-based stratification at predefined intervals (end of induction and post-consolidation) is the cornerstone of risk assignment and is applied far more frequently than MRD-directed immunotherapy in refractory disease. Key decision time points are: (i) end of induction (2–4 weeks), where MRD ≥ 10^−4^ often triggers early intensification with blinatumomab; and (ii) post-consolidation (2–3 months or after three blocks), which guides definitive risk stratification and allo-HCT planning. Longitudinal/serial monitoring every three months during maintenance mainly aims to detect molecular relapse. In contrast to pediatric protocols [10,65], therapy de-escalation based on deep MRD negativity remains investigational and limited in adults, reserved mainly for selected standard-risk patients with sustained ultra-deep (10^−6^) NGS negativity. During longitudinal monitoring, especially post-immunotherapy, repeated MFC is essential not only for MRD quantification but also for evaluating persistence or loss of therapeutic targets (CD19/CD22)

Table 2 summarizes key MRD timing, thresholds, and actions.

### 4.1. Frontline Multi-Agent Chemotherapy: Timing, Thresholds, and Escalation

Standardized MRD assessment is essential during frontline multi-agent chemotherapy (pediatric-inspired regimens +/− targeted agents)

The 2024 ELN recommendations emphasize prospective MRD assessment at early timepoints, 2–3 months from diagnosis (after first consolidation or three blocks of therapy) to identify chemotherapy resistance. The commonly applied threshold of 0.01% (10^−4^) remains the benchmark for clinical decision-making, with each log increment associated with shortened time to relapse (median not reached or >12 months for MRD < 0.01% vs. 7.6 months for MRD > 0.01% but ≤0.1% vs. 4.9 months for MRD > 0.1%) [5].

The 2025 US expert panel similarly recommends MRD assessment after induction (2–4 weeks) and consolidation (12–16 weeks), followed by surveillance every 3–4 months for at least 2–3 years, with closer monitoring for persistent or recurrent MRD.

Data from the GMALL group demonstrate a median interval of approximately four months between molecular recurrence (ASO-qPCR) and overt relapse, supporting surveillance intervals ≤3 months during high-risk periods. Most relapses occur within the first three years [66,67].

In patients harboring high-risk molecular abnormalities (such as IKZF1 deletions with or without CDKN2A/B loss [known as IKZF1-plus genotype], Ph-like signatures, or TP53 mutations), achieving MRD negativity, even at ultrasensitive levels like 10^−6^ via NGS, may not fully mitigate the inherent aggressive disease biology. Although deep MRD clearance holds potential [59,68], both sets of guidelines urge caution when considering omission of allo-HCT in these cases, citing insufficient evidence, and endorse it as the standard consolidation approach pending robust data from larger trials to validate safe deferral.

### 4.2. Persistent MRD After Chemotherapy

Persistent MRD post-consolidation signals resistance and is a key escalation trigger.

In B-ALL, the BLAST study demonstrated high MRD clearance rates with blinatumomab in patients with persistent MRD ≥ 10^−3^, with molecular responders achieving favorable relapse-free survival and frequently proceeding to HSCT [2,69,70]. The ECOG-ACRIN E1910 trial extended this benefit: adding blinatumomab to consolidation in MRD-negative BCP-ALL improved 3-year OS (85% vs. 68%; HR 0.41, *p* = 0.002) [71], shifting the paradigm toward immunotherapy even in negativity. This finding, together with excellent outcomes in deep responders treated with chemotherapy-free regimens such as ponatinib plus blinatumomab, supports the growing safety of deferring allo-HCT in selected standard-risk patients who achieve sustained deep MRD negativity, particularly when documented by high-sensitivity NGS.

In contrast, the Alliance A041501 trial found inotuzumab improved early MRD negativity but not three-year event-free survival (HR 0.93, *p* = 0.65), with increased hepatotoxicity; highlighting that molecular clearance does not always translate to survival benefit if offset by toxicity [72].

Undetectable NGS-MRD post-consolidation correlates with superior OS. In one retrospective cohort of 84 Ph-negative B-ALL patients in morphologic remission, 38% were NGS-positive despite MFC negativity, with NGS negativity linked to lower relapse risk and longer survival [62].

In Ph+ ALL, chemotherapy-free or TKI-immunotherapy regimens (dasatinib + blinatumomab; ponatinib + blinatumomab), early MRD negativity by NGS (10^−6^) or qPCR at 2–3 months correlates with improved survival (4-year disease-free survival (DFS) > 80% in dasatinib + blinatumomab; three-year RFS > 90–95% in ponatinib + blinatumomab), supporting potential transplant deferral in selected patients achieving sustained deep responses [62,73].

Risk refinement is increasingly integrating MRD with genomic features. Some studies proposed a combined model in adult Ph-negative B-ALL that incorporates both MRD response and *IKZF1* deletion status. Patients are classified as standard-risk when MRD-negative and *IKZF1* wild-type, intermediate-risk when either MRD-positive or carrying an *IKZF1* deletion, and high-risk when both MRD positivity and IKZF1 deletion are present. This integrated approach more accurately predicts relapse risk and may improve allo-HCT selection, particularly in genetically adverse B-ALL subtypes where persistent MRD is associated with poor outcomes [74].

Both guidelines recommend MRD-directed therapy for persistent or recurrent disease. The 2025 US expert panel takes a particularly pragmatic approach, recommending intervention for any detectable MRD level provided there is high confidence the result represents true leukemic disease (rather than background noise), with blinatumomab favored as first-line for CD19-naïve B-ALL patients. The 2024 ELN guidelines similarly advocate escalation for persistent MRD (typically at the 10^−4^ threshold or higher), but emphasize that low-level positivity (10^−5^–10^−6^) should be interpreted cautiously and ideally confirmed with orthogonal methods before triggering therapy changes.

### 4.3. MRD and Allogeneic HSCT: Pre- and Post-Transplant Risk

#### 4.3.1. Pre-HSCT MRD

Pre-transplant MRD negativity is one of the strongest predictors of post-transplant outcome [75]. In an international cohort (616 patients) low-level pre-HSCT MRD yielded outcomes similar to negativity, while high burden (>0.1%) predicted inferior survival [76]. NGS-defined MRD > 10^−4^ prior to transplant has been associated with a 7.7-fold increased relapse risk [77]. Burden, not mere positivity, matters. Whether eradication below 10^−4^ or 10^−6^ improves outcomes remains unresolved.

#### 4.3.2. Post-HSCT MRD

Post-transplant MRD carry even greater prognostic weight. Detectable qPCR-MRD at day +100 has been associated with relapse risks approaching 80% compared with approximately 7% in undetectable cases [78]. NGS positivity post-HSCT often precedes relapse by approximately three months [79]. PB NGS monitoring appears feasible and correlates with BM findings in selected cohorts [13,64].

Guideline recommendations reflect this evolving landscape. The 2025 US expert panel supports deferring allo-HCT in standard-risk patients (including Ph+ ALL without *IKZF1* genotype) who achieve sustained MRD negativity (by NGS for IG/TR and/or PCR for *BCR::ABL1* in Ph+ cases) within 3 months of frontline therapy, emphasizing the prognostic importance of initial deep NGS negativity (down to 10^−6^) and the ‘any detectable’ approach, while high-risk patients with deep negativity (1 × 10^−6^) require individualized decisions weighing relapse risk against transplant toxicity, with serial monitoring if HCT is deferred in contrast, the 2024 ELN guidelines adopt a more conservative approach, primarily reserving HCT for poor MRD responders (e.g., persistent MRD > 0.01% after three blocks), due to limited prospective data at deeper levels.

Early deep negativity may allow omission in select standard-risk cases per protocol, but HCT remains standard for younger patients converting to negativity, with dose-reduced or non-transplant strategies considered for older/high-risk patients. These differences highlight ongoing debate on balancing relapse prevention against transplant-related morbidity, particularly in high-risk molecular subsets with deep responses.

### 4.4. MRD in Relapsed/Refractory Disease

MRD status has driven regulatory approvals and outcome prediction for targeted therapies in R/R B-ALL. Blinatumomab was approved for MRD-positive disease based on the BLAST trial, where clearance (78% after 1st cycle (88/113 evaluable patients) correlated with improved RFS (23.6 months vs. 5.7 months (*p* = 0.002)) and OS (38.9 months vs. 12.5 months (*p* = 0.002)) [69,80]. In INO-VATE, inotuzumab ozogamicin achieved higher rates of MRD-negative CR/CRi (46% overall; 78% of responders [68/87 evaluable] achieved MRD < 0.01% by MFC) compared with standard chemotherapy (28% of responders), with MRD-negative responders demonstrating superior outcomes including longer remission duration (median 4.6 months vs. 3.1 months; HR 0.55, *p* = 0.03), improved progression-free survival (PFS) (median 5.0 months vs. 1.8 months; HR 0.45, *p* < 0.001), and better OS, particularly among those proceeding to allo-HCT (median OS 12.6 months vs. 7.1 months in non-transplanted CR/CRi patients; *p* = 0.007) [3,81].

CAR-T therapy induces MRD negativity in approximately 80% of adult patients in meta-analytic data, though durability varies; post-CAR MRD positivity often prompts HSCT or investigational approaches [82,83]. In an analysis of children and young adults with relapsed/refractory B-cell ALL who received tisagenlecleucel, detectable MRD at any level by NGS for IG/TR independently predicted for worse event-free survival (EFS) and OS. The median EFS for patients with detectable MRD 3 months after CAR T-cell therapy was only 5.8 months vs. not estimable in those who were NGS MRD negative (*p* < 0.0001). Given the poor outcomes of patients with persistent MRD after CAR T-cells, allo-HCT (or other MRD-directed therapies) should be considered for these patients, whereas the role of consolidative HCT for MRD-negative patients is less clear [84].

In Ph+ B-ALL, MRD re-emergence may indicate acquisition of *ABL1* kinase domain mutations (e.g., *T315I*), although detection sensitivity is limited at low transcript levels [85]. Combination strategies such as blinatumomab plus ponatinib aim to eradicate MRD and suppress resistant clones [86,87].

Both ELN and US panels recommend MRD-directed therapy for persistent or recurrent disease, favoring blinatumomab in CD19-naïve B-ALL and reserving inotuzumab, CAR-T, or investigational approaches for subsequent lines.

## 5. Ultra-Low-Level MRD (10^−5^–10^−6^): Prognostic Significance and Current Actionability in B-ALL

The advent of NGS-based assays capable of reliably detecting MRD at 10^−5^ to 10^−6^ has redefined prognostic thresholds in adult B-ALL, yet whether these ultra-low levels warrant therapeutic intervention remains one of the most debated questions in the field.

Several key studies have begun to address this. A study by MD Anderson Cancer Center evaluated NGS MRD at 10^−6^ sensitivity in 214 newly diagnosed Ph-negative B-ALL patients treated with hyper-CVAD-based regimens; patients achieving negativity at 10^−6^ after induction or consolidation had 5-year RFS exceeding 90% and very low relapse rates, even among those with adverse cytogenetics [88]. A subsequent 2025 analysis by the same group (187 patients) showed that early NGS negativity at 10^−6^ was the strongest predictor of long-term disease-free survival, outperforming traditional risk factors and supporting potential therapy de-escalation in deep responders [89].

A study pooled low-level NGS and ddPCR data from multiple adult ALL cohorts and found that persistent MRD between 10^−5^ and 10^−4^ carried intermediate relapse risk (cumulative incidence 30–40% at 3 years), while levels <10^−5^ approached the excellent outcomes of MRD-negativity by the assay’s sensitivity level [60].

Emerging prospective protocols are testing actionability directly. The GMALL group has incorporated NGS-guided de-escalation arms in standard-risk B-ALL patients achieving sustained 10^−6^ negativity post-consolidation, aiming to reduce treatment intensity while preserving cure rates [90]. Similarly, pediatric trials such as COG AALL1731 (NCT03914625) have incorporated NGS-based MRD assessment to refine risk stratification and explore therapy intensification with blinatumomab in higher-risk standard-risk patients, with emerging data suggesting that deep NGS negativity may identify candidates for reduced intensity in future pediatric-inspired protocols.

Despite these signals, prospective randomized evidence that intervening at 10^−5^–10^−6^ improves survival compared with observation is still lacking. Current guidelines reflect this uncertainty: the 2025 US panel favors NGS-driven de-escalation in standard-risk patients with sustained negativity at 10^−6^, while the 2024 ELN maintains the 10^−4^ threshold as the primary action point and cautions against overtreatment based solely on ultra-low positivity. Until mature randomized data emerge, clinical decisions at these levels should remain individualized, contextual, and ideally made within prospective trials.

## 6. Future Directions

International harmonization of MRD methodologies is an urgent priority. The EuroClonality-NGS consortium has developed standardized amplicon-based NGS approaches for IG/TR MRD detection that achieve sensitivities approaching 10^−6^ with high reproducibility across European laboratories. These efforts provide an important non-commercial counterpart to clonoSEQ and support future international harmonization of NGS-based MRD reporting [35,51].

Despite this progress, substantial differences persist. US practice remains largely centered on clonoSEQ, while European efforts prioritize EuroClonality-NGS and EuroMRD pipelines. As sensitivity continues to increase, these divergent frameworks risk widening variability in MRD interpretation across centers and trials.

Emerging technologies hold further promise. Single-cell sequencing could resolve clonal architecture and lineage plasticity with unprecedented detail, while cell-free DNA assays may enable minimally invasive, serial plasma-based monitoring. Both remain in early validation stages for ALL [91].

Finally, equitable access to high-sensitivity MRD assessment remains a major global challenge. The cost of NGS testing, the need for advanced sequencing infrastructure and bioinformatics expertise, and prolonged turnaround times limit adoption in many low- and middle-income countries (LMICs), where even standardized multiparameter flow cytometry may be inconsistently available. A recent systematic review underscored the scarcity of MRD data from LMICs, raising concern that widening use of NGS in high-resource settings may further exacerbate outcome disparities. Efforts to address this gap include development of lower-cost sequencing platforms, cloud-based bioinformatics pipelines, and multicenter harmonization initiatives such as EuroClonality-NGS and EuroMRD quality assurance programs aimed at scalable, reproducible implementation across diverse healthcare systems [16,17,51].

Over the coming decade, the challenge will not be detecting smaller quantities of disease, but determining when deeper detection truly changes outcomes, and ensuring that precision does not outpace proof.

## 7. Conclusions

MRD has become central to risk stratification and therapeutic decision-making in adult ALL, consistently predicting outcome and increasingly guiding escalation or de-escalation strategies. The adoption of high-sensitivity NGS has refined prognostic discrimination beyond the traditional 10^−4^ threshold, revealing biologic heterogeneity at ultra-low levels. However, improved detection does not automatically equate to improved survival. Whether intervention based solely on MRD levels between 10^−5^ and 10^−6^ alters long-term outcomes remains unresolved. MRD interpretation must therefore be contextual, rather than applied as a uniform numeric trigger. As the field advances, the central challenge will be aligning technical sensitivity with evidence-based actionability, ensuring that precision enhances care without driving overtreatment.

## Figures and Tables

**Table 1:** Comparison of MRD detection modalities in adult ALL.

Modality	Analytic Sensitivity	Quantification Approach	Key Strengths	Key Limitations/Vulnerabilities	Preferred Clinical Contexts/Notes	Guideline Preference (2024 ELN vs. 2025 US)
Multiparameter Flow Cytometry (MFC)	10^−4^ (routine); up to 10^−5^ (optimized)	percentage of aberrant blasts in current sample (snapshot)	Rapid, accessible, no patient-specific setup; EuroFlow panels standardized, provides phenotypic information relevant to targeted treatment	Antigen modulation/loss (e.g., CD19/CD22 post-immunotherapy); lineage switch; operator-dependent; specimen quality sensitive	Urgent settings, resource-limited; post-CD19 therapy with adapted panels; simultaneous target assessment	Both support routine use at 10^−4^ benchmark; ELN more conservative (≥10^−4^ considered “molecular failure” triggering escalation); US allows intervention for any detectable MRD if confident true disease
Quantitative PCR (qPCR)	10^−4^ to 10^−5^	Log reduction from baseline or normalized ratio to reference gene/target	Molecular specificity (fusions like BCR::ABL1 or IG/TR ASO); stable under phenotypic changes; ddPCR refinements improve low-level performance	Labor-intensive setup (4–6 weeks); clonal evolution false negatives; high rate of POS-NQ/PNQ at low levels; target-dependent applicability	Ph+ ALL (BCR::ABL1 monitoring); fusion-positive cases; orthogonal to MFC post-immunotherapy	ELN favors for most cases (esp. fusions/IG-TR); ddPCR refinements noted. US accepts but favors NGS for deeper sensitivity
Next-Generation Sequencing (NGS) (e.g., clonoSEQ)	Up to 10^−6^ or lower	Absolute or relative clone frequency (multi-clone tracking)	Highest sensitivity; tracks multiple clones; less clone-biased; continuous quantification; possible PB monitoring	Cost, turnaround (7–10 days), bioinformatics needs, input DNA requirements; background noise	Preferred for deep assessment in B-ALL; early negativity for de-escalation/transplant deferral; PB monitoring potential	2025 US: Preferred for B-ALL decision-making (any detectable at 10^−6^ sensitivity, PB NGS allowed after BM negativity); 2024 ELN: Mainly for molecular subtypes, not routine IG/TR (remains BM-centric)

**Table 2:** Key MRD timing, thresholds, and actions.

Timepoint	Common Threshold for Actionability	Prognostic Implication (General)	Recommended Action (2024 ELN)	Recommended Action (2025 US)	Notes/Subtype Considerations	Primary Therapeutic Consequence in Adults
Post-Induction (2–4 weeks)	≥10^−4^ (0.01%)	Persistent MRD → higher relapse risk	Assess; persistent → consider escalation (e.g., immunotherapy)	Assess; NGS preferred for deeper insight; early deep negativity (10^−6^) may support de-escalation in select standard-risk/Ph+	Early NGS negativity increasingly used to identify very low-risk patients (US panel emphasis)	Early intensification (e.g., add blinatumomab)
Post-Consolidation (2–3 months/3 blocks)	≥10^−4^	Strongest escalation trigger; >1 log rise shortens time to relapse	Often → allo-HCT +/− targeted therapy if persistent	NGS negativity → potential HCT deferral in standard-risk; intervene for any detectable MRD if confident it is true disease	US panel: intervene at any level if high confidence; ELN uses 10^−4^ as primary action point	Definitive risk assignment + allo-HCT planning
Maintenance/Surveillance	Detectable (any level, esp. rising)	Molecular relapse precedes overt relapse (~3–4 months)	Every 3 months; persistent/recurrent → intervention	Every 3–4 months for 2–3 years; closer (1–2 months) if persistent; PB NGS feasible after BM negativity	PB monitoring preferred by US panel once deep BM negativity confirmed; ELN remains BM-centric	Detection of molecular relapse → pre-emptive intervention
Pre-HCT	>10^−4^ (esp. >10^−3^)	High MRD → inferior post-HCT outcomes	Eradicate if possible; low-level similar to negative	Burden matters; defer in sustained negativity (standard-risk); individualize high-risk deep negativity	NGS/PCR negativity strongest predictor; US panel supports deferral in standard-risk deep responders	Decision to proceed with or defer HCT
Post-HCT	Detectable (esp. day +100)	High relapse risk	Monitor; pre-emptive intervention	NGS positivity often precedes relapse by ~3 months; intervene if confident true MRD	PB NGS monitoring emerging (US panel); pre-emptive therapy (e.g., blinatumomab) increasingly used	Pre-emptive immunotherapy or DLI

## Data Availability

No new data were created or analyzed in this study. Data sharing is not applicable to this article.
